# Cross-linking of IgGs bound on circulating neutrophils leads to an activation of endothelial cells: possible role of rheumatoid factors in rheumatoid arthritis-associated vascular dysfunction

**DOI:** 10.1186/1476-9255-10-27

**Published:** 2013-07-31

**Authors:** Emmanuelle Rollet-Labelle, Myriam Vaillancourt, Louis Marois, Marianna M Newkirk, Patrice E Poubelle, Paul H Naccache

**Affiliations:** 1 Départements de Microbiologie-Infectiologie et Immunologie et de Médecine, Centre de recherche en rhumatologie et immunologie, Centre de recherche du CHU de Québec, Université Laval, Québec, QC, Canada; 2 Départements de Microbiologie-Infectiologie et Immunologie et de Médecine, Faculté de médecine, Université Laval, Québec, QC, Canada; 3 Department of Medicine, Division of Rheumatology, Research Institute of McGill University Health Centre, McGill University, Montreal, QC, Canada

**Keywords:** Rheumatoid factors, Human neutrophils, FcγRs, Endothelial cells, Vascular damage

## Abstract

**Background:**

Rheumatoid arthritis is characterized by the presence of circulating auto-antibodies, including rheumatoid factors, which recognize the Fc portion of IgGs. The neutrophil is the most abundant circulating leukocyte and it expresses high levels of FcγRs on its surface. The aim of the present study was to examine the capacity of circulating human neutrophils to be activated by rheumatoid factors and the consequences of these events on endothelium.

**Methods:**

Neutrophil-bound IgGs were cross-linked with anti-human IgGs to mimick the presence of circulating rheumatoid factors and FcγRs-dependent signalling events and functions were examined. The IgG and IgM composition of rheumatoid factors isolated from the serum of RA patients was characterized. Adhesion of neutrophils to endothelial cells was quantified in response to the addition of rheumatoid factors.

**Results:**

Cross-linking of IgGs bound on neutrophils leads to FcγRs-dependent tyrosine phosphorylation, mobilisation of intracellular calcium and the extracellular release of superoxide anions and lysozyme. Incubation of endothelial cells with the supernatant of activated neutrophils increases ICAM-1 expression and IL-8 production by endothelial cells. Finally, rheumatoid factors enhance neutrophil adhesion to endothelial cells.

**Conclusions:**

Our results show that activation of neutrophils’ FcγRs by rheumatoid factors could participate in rheumatoid arthritis-associated vascular damage.

## Background

Rheumatoid arthritis (RA) is a chronic auto-immune inflammatory disease whose origin remains poorly understood. It is characterized by infiltration of leukocytes (mostly neutrophils) in the joints and a proliferation of the lining synovial cells often leading to the irreversible destruction of cartilage and bone. RA also has systemic manifestations [1-3]. Vascular inflammation is a severe complication of RA and patients with RA show an increased risk for cardiovascular events compared with the general population [4-6].

There is long-standing evidence for the presence of increased titers of auto-antibodies in the serum of RA patients. The best known are rheumatoid factors (RFs) and anti-cyclic citrullinated peptide (anti-CCP) antibodies the quantitation of which is currently used for clinical diagnostic purposes [7]. Results obtained with animal models point to a crucial role for these antibodies in the pathology of arthritis [8,9]. Rheumatoid factors recognize the Fc portion of IgGs. They are found in up to 80% of the sera of RA patients and represent a diagnostic and prognostic marker for RA even though they are detected in other auto-immune diseases and systemic infections [10]. RFs can enhance immune complex formation by interacting with circulating free IgGs, which are present at high concentrations (10 mg/ml) in human serum [11].

FcγRs specifically recognize the Fc portion of IgGs. Neutrophils, the most abundant circulating leukocytes, express at their surface high amounts of a specific and unique combination of FcγRs, namely FcγRIIa and FcγRIIIb. These receptors play key roles in neutrophil phagocytosis as they participate in the recognition and phagocytosis of pathogens opsonized with IgGs. Neutrophils also possess a battery of cytotoxic enzymes that can be delivered within intracellular phagosomes to effect killing but may be inadvertently released extracellularly with deleterious consequences for the surrounding tissues [12]. FcγRs can also recognize naturally occurring immune complexes (ICs) and they play key roles in autoimmunity as they participate in the clearance of immune complexes which are abundant in the joint and circulation of RA patients [13,14]. Recently, an association of FcγRIIIb copy number with the susceptibility to RF-positive RA was reported [15].

Since neutrophils are the major cells which infiltrate the synovial fluid and tissues [16] in RA, the activation of FcγRs on neutrophils is likely to play an important role in the chronic nature and the severity of the disease. Several murine models have been developed to test this hypothesis and to define the specific contribution of the different FcγRs [17,18]. Mice deficient in the common γ-signalling chain of FcγRs are not susceptible to arthritis induction; in contrast, the lack of the inhibitory receptor FcγRIIb exacerbates collagen-induced arthritis in susceptible mice [19]. However, as mouse and human FcγRs differ in their expression and cellular distribution, it is difficult to extrapolate results observed in mouse models to human diseases. Nevertheless, FcγRIIa has been implicated in RA as human FcγRIIa transgenic mice are susceptible to collagen-induced arthritis [19,20]. Neutrophil-selective transgenic expression of human FcγRIIa and FcγRIIIb in this model indicates that these two receptors play non-redundant roles [13]. Belostocki et al. observed that FcγRIIa expression on circulating neutrophils was decreased during a longitudinal follow-up of RA patients receiving four infliximab infusions [21].

These data, together with the known role of neutrophils in tissue injury in IgG-mediated diseases and atherosclerosis, indicate that a detailed examination of the role of RFs on neutrophils and their potential implication in RA-associated vascular damage is warranted [22,23]. Whereas most of the neutrophil studies examine their roles outside the blood vessels, circulating neutrophils can have a pathological function in the systemic inflammatory condition that is characteristic of RA. In RA, circulating ICs first deposit in vasculature and FcγRIIIb plays an important role in slowing down neutrophils leading to adhesion [24-26]. Neutrophils can then signal cytotoxic functions through, among others, FcγRs thus promoting tissue damage [27]. These data contributed to the elaboration of our hypothesis that IC and RF-containing IC can activate FcγRs on circulating neutrophils and participate in the development of RA-associated endothelium damage.

## Methods

### Antibodies and reagents

#### Antibodies

The anti-phosphotyrosine antibody (4G10, catalog no.05-321X) was purchased from Millipore (Billerica, MA). Horseradish peroxidase-labelled sheep anti-mouse IgGs (catalog no.NXA931) was obtained from GE Healthcare (Uppsala, Sweden). The anti-actin antibody (sc-1616) was obtained from Santa-cruz Biotechnology (Santa Cruz, CA). The anti-ICAM1 antibody (catalog no.BBA4) was obtained from R&D systems (Minneapolis, MN). Horseradish peroxidase-labelled anti-human IgGs, anti-human IgMs and anti-goat IgGs, FITC-labelled anti-human IgGs (catalog no.109-096-097) and anti-mouse IgGs (catalog no.115-096-071) and the rabbit anti-human IgGs (H + L) (catalog no.309-005-082) and rabbit anti-human IgGs, Fc fragment specific (catalog no.309-005-008) were obtained from Jackson ImmunoResearch (West Grove, PA). The anti-FcγRIIIb Pelicluster (clone CLB-FcR-gran/1) (catalog no. M1389) was purchased from Sanquin (Amsterdam, The Netherlands). The anti-FcγRIIa antibody (clone IV.3) was purified from ascites of mice inoculated with hybridoma HB-217 obtained from the American Type Culture Collection (Manassas, VA).

#### Reagents

Calcein AM and Fura-2 AM were obtained from Invitrogen Life technologies (Burlington, ON). Cytochrome *c* was purchased from EMD chemicals (Mississauga, ON). Dextran T-500 and *Micrococcus lysodeikticus* were purchased from Sigma (Oakville, ON). Western lightning chemiluminescence plus was obtained from Perkin Elmer (Boston, MA). Ficoll-Paque and Hepes were obtained from Wisent (St-Bruno, QC).

### Isolation and stimulation of neutrophils

The collection of the blood was done with an appropriate consent form and approval of Laval University ethics committee. Neutrophils were aseptically isolated from healthy donors as previously described [28]. They were resuspended at 20x10^6^ cells/ml in Mg^2+^-free HBSS containing 1.6 mM of CaCl_2_. Autologous platelet-poor plasma (PPP) was prepared by centrifugation of whole plasma at 3000xg for 10 minutes. To restore the physiologic amounts of bound IgGs on isolated cells, neutrophils were incubated for 20 minutes on ice with 10% autologous PPP followed by a 2 minutes centrifugation at 600xg. The neutrophils’ FcγRs were cross-linked by incubation with rabbit anti-human IgGs (20 μg/ml, final concentration) or with RFs (500 μg/ml) at 37°C for the times indicated in the legends of the figures. For FcγRs blocking experiments, antibodies IV.3 (2 μg/ml) (anti-FcγRIIa) and Pelicluster (4 μg/ml) (anti-FcγRIIIb) were added 10 minutes before incubation with autologous PPP. For the preparation of neutrophil supernatants, IgG were cross-linked with rabbit anti-human IgG antibody for 30 minutes at 37°C in the presence of 0.5% BSA. Neutrophils were then centrifuged (13000xg, 1 minute) and supernatants were collected and re-centrifuged before filtration on 0.22 μM membranes.

### Electrophoresis and immunoblotting

Proteins were separated by SDS-PAGE on 7.5-15% or 10% acrylamide gels and transferred on PVDF membranes. They were then analyzed by immunoblotting as described in the legends of the figures.

### Calcium mobilisation

Neutrophils were pre-incubated with 1 μM fura-2 AM for 30 minutes at 37°C in the presence of 0.5% BSA. The extracellular probe was removed by centrifugation and the cells were resuspended in HBSS at 5x10^6^ cells/ml and stimulated as described in the legend of the figures. Fluorescence was monitored in a fluorescence spectrophotometer (Fluorolog-SPEX from Jobin Yvon Inc., Edison, NJ) using two excitation wavelengths of 340 and 380 nm and an emission wavelength of 510 nm. The ratio of fluorescence values obtained at 340 and 380 nm was used as a measure of the intracellular levels of free cytoplasmic calcium.

### Degranulation

The extent of lysozyme release was assessed by adding 100 μl of neutrophil supernatants to 900 μl of a 0.25 mg/ml *Micrococcus lysodeikticus* solution prepared in a 0.1M PO_4_ buffer. The loss of absorbance was then monitored at 450 nm for 5 minutes and the rate of decrease of the absorbance provided a measure of the lysozyme activity present in the supernatants. The slopes were normalized to that of a cell lysate obtained by lysing the cells with 0.1% Triton X-100.

### Superoxide production

Superoxide production was measured using the cytochrome *c* reduction assay. The absorption of cytochrome *c* was monitored at 550 nm and 540 nm and the amount of superoxide anions produced was calculated from the difference between the OD at the two wavelengths.

### Culture and stimulation of HUVECs

Human umbilical vein endothelial cells (HUVECs) were obtained from Lonza (Basel, Switzerland). They were cultured in a humid atmosphere containing 5% CO_2_ in EGM medium complemented with bovine brain extract as indicated by the company and used at passages 2–6. For the measurement of ICAM-1 expression and IL-8 production, HUVECs were seeded at 13x10^4^cells/well in 6-well plates, grown for 24 hours and stimulated for 24 hours with the supernatants of control or IgGs-cross-linked human neutrophils diluted 1:1 in EGM complete medium. After stimulation, the supernatants of the HUVECs were collected and the cells were stimulated for another 24 hours with a new aliquot of the same supernatants of control or IgGs cross-linked human neutrophils diluted 1:1 in EGM complete medium following which the supernatants of the HUVECs were collected again.

### Adhesion of neutrophils to endothelial cells

To obtain confluent monolayers, endothelial cells were seeded at 4–5 000 cells/well in 96-well plates and grown for 72–96 hours. Isolated neutrophils (20x10^6^/ml) were labeled in HBSS containing 5 μM calcein AM for 30 minutes at 37°C in the dark. PPP (10%, final concentration) was added to the neutrophil suspension for 20 minutes on ice following which the neutrophils were centrifuged and resuspended (5x10^6^/ml) in HBSS containing 0.8 mM MgCl_2_. Neutrophils (25x10^4^ cells) were then added to the 96-well plates containing the confluent HUVEC monolayers and stimulated with anti-human IgGs antibody for 30 minutes or RFs for two hours at 37°C in a humid atmosphere containing 5% CO_2_ in the dark. Non-adherent cells were washed away with cold PBS and the fluorescence of the plates resulting from the adhesion of calcein-labelled neutrophils to HUVECs was monitored at 485/530 nm (excitation/emission).

### Preparation of rheumatoid factors

The collection of the blood was done with an appropriate consent form and approval of McGill University ethics committee. Selected sera from RF positive RA patients were pooled and centrifuged at 10,000 rpm for 15 minutes to remove any particulate matter. The supernatant was then made to a final concentration of 5% polyethylene glycol 6000 (by adding an equal volume of 10%) and mixed well. After 16 hrs at 4°C, the precipitates were collected by centrifugation for 15 minutes at 10,000 rpm. These precipitates were resuspended in PBS/azide to the original volume of the serum or plasma (and thus diluting out the PEG). The semi-purified RF-containing solutions were then stored at −20°C in a 50% glycerol solution to prevent damage from freeze thawing. Pentameric IgM precipitates at 5% PEG, whereas free IgG requires 10-12% PEG in order to precipitate from solution, thus the complexes collected using 5% PEG are primarily those that result from the IgM RF recognition, although IgG RF complexes may also be present.

## Results

**Figure 1:**
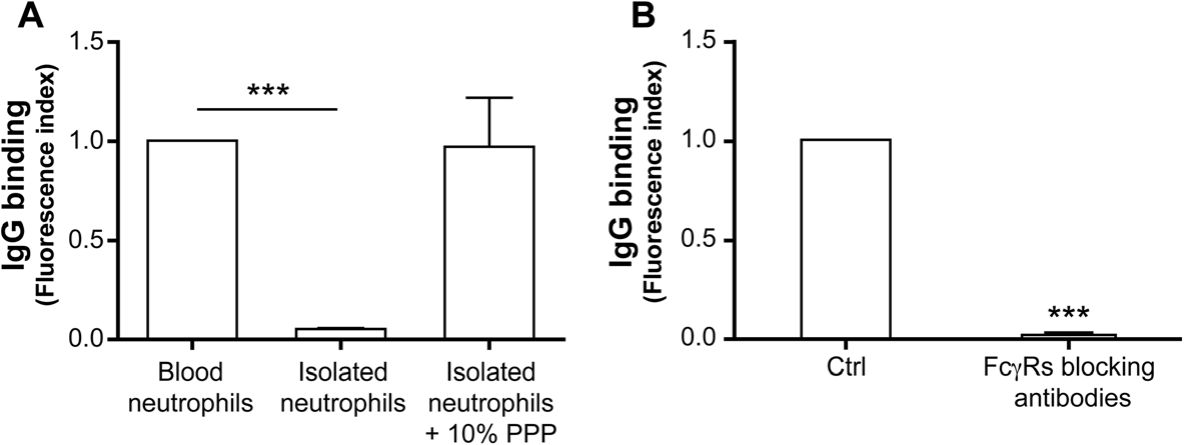
**Circulating blood neutrophils constitutively possess IgGs bound to their FcγRs. A**: For the measurement of IgGs present on neutrophils in whole blood, erythrocytes were lysed by dilution of 900 μl of whole blood in 11 ml of an NH_4_Cl buffer containing 138.3 mM NH_4_Cl, 21.5mM Tris, pH 7.4 as previously described [30]. After the blood became translucent, leukocytes were recovered by a 5 minute centrifugation at 800 g. The amount of IgGs bound on neutrophils was measured by flow cytometry using FITC-labeled anti-human IgG antibodies. The same measurement was performed on isolated neutrophils incubated with or without 10% autologous PPP for 20 minutes. **B**: Neutrophils were pre-incubated with blocking FcγRs antibodies before incubation with PPP and analysis as in **A**. Results are expressed as mean +/− SEM of five independent experiments. *** P < 0.0005 (one-sample t test).

As RFs recognize the Fc portion of IgG, they can form IC with circulating IgGs and deliver IgGs to neutrophils potentially leading to the activation of FcγR-dependent signalling pathways and functions. Alternatively, RFs may directly recognize surface-bound IgGs, cross-link them and, in so doing, activate neutrophils. Indeed, because of the high concentration of IgGs in plasma (10 mg/ml), neutrophils carry surface-bound IgGs in spite of the low affinity of FcγRs for monomeric IgGs. Although IgGs are dissociated from neutrophils after several washing steps [29], we postulated that incubation of isolated neutrophils with autologous plasma could restore the level of bound IgGs to that physiologically present on circulating neutrophils. We measured the amount of IgGs on freshly isolated neutrophils incubated with 10% of autologous PPP and compared it with the amount of IgGs measured on blood neutrophils (Figure 1A). The data confirm the loss of surface-bound IgGs on isolated neutrophils and indicate that incubation with 10% PPP leads to a recovery of an amount of bound IgGs similar to that present on circulating neutrophils. Furthermore, we also showed that plasma IgGs specifically bind to FcγRs as blocking these receptors with a combination of specific blocking antibodies to both anti-FcγRIIa and anti-FcγRIIIb abrogated the binding of IgGs to isolated neutrophils (Figure 1B).

**Figure 2:**
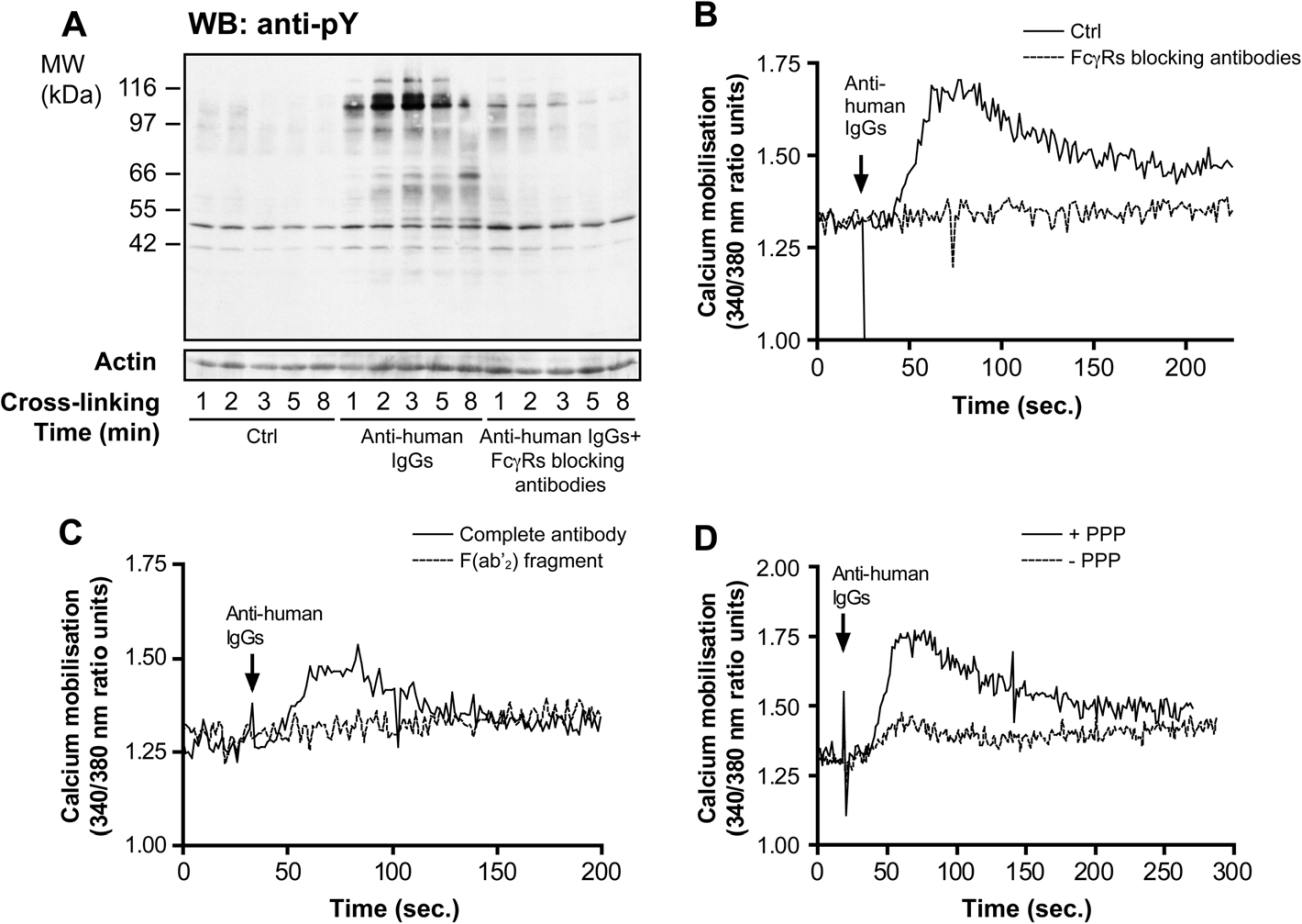
**Cross-linking of IgGs bound to human neutrophils leads to FcγR**-**dependent tyrosine phosphorylation and calcium mobilisation. A**: Isolated neutrophils were incubated with 10% autologous PPP with or without FcγRs blocking monoclonal antibodies as described in Methods and Figure 1. Cross-linking of surface-bound IgGs was achieved by the addition of anti-human IgG antibodies. The reaction was stopped by transferring aliquots of the neutrophil suspensions directly in the same volume of boiling 2X concentrated modified Laemmli’s buffer [33]. Whole cell lysates (2.5x10^5^ cells in each lane) were analysed by SDS-PAGE and immunoblotted with anti-phosphotyrosine antibody 4G10 or anti-actin antibody (loading control). **B**: Isolated neutrophils were preincubated with Fura2-AM, followed by an incubation with or without FcγRs blocking antibodies and with 10% autologous PPP. Anti-human IgG antibodies were added and fluorescence was monitored as described in Methods. **C**: Cross-linking of surface-bound IgGs was achieved by the addition of either complete anti-human IgG antibodies or F(ab’)_2_ fragment of anti-human IgG antibodies. **D**: Neutrophils were incubated with or without PPP before cross-linking of surface bound IgGs. Results are representative of, at least, three independent experiments.

To test the hypothesis that the presence of RFs can lead to the activation of circulating neutrophils *via* their FcγRs, we used commercially available rabbit antibodies with the same antigenic characteristics as RFs, i.e. anti-human IgG antibodies. We first examined whether these antibodies could stimulate FcγR-dependent signalling pathways in human neutrophils. We previously showed that the specific engagement of FcγRIIa or FcγRIIIb (cross-linking using monoclonal antibodies specific for FcγRIIa and FcγRIIIb) leads to an increase of the tyrosine phosphorylation pattern of whole neutrophils lysates and to an intracellular mobilisation of calcium [31,32]. Figure 2 indicates that the addition of the anti-human IgG antibodies led to an enhancement of the FcγR-dependent tyrosine phosphorylation profile (see the proteins in the 60 to 120 kDa range) as well as to an increase in the level of free cytoplasmic calcium in isolated neutrophils pre-incubated with PPP. Both responses were decreased if the F(ab’)_2_ fragment of the anti-human IgG antibodies rather than the complete antibodies were used (Figure 2C and data not shown). This result indicates that these FcγR-dependent signalling pathways are in part activated by direct binding of anti-human IgG antibodies to free FcγRs. Furthermore, these responses were also decreased when neutrophils were not pre-incubated with PPP (Figure 2D and data not shown) confirming the capacity of anti-human antibodies to cross-link surface-bound IgGs.

**Figure 3:**
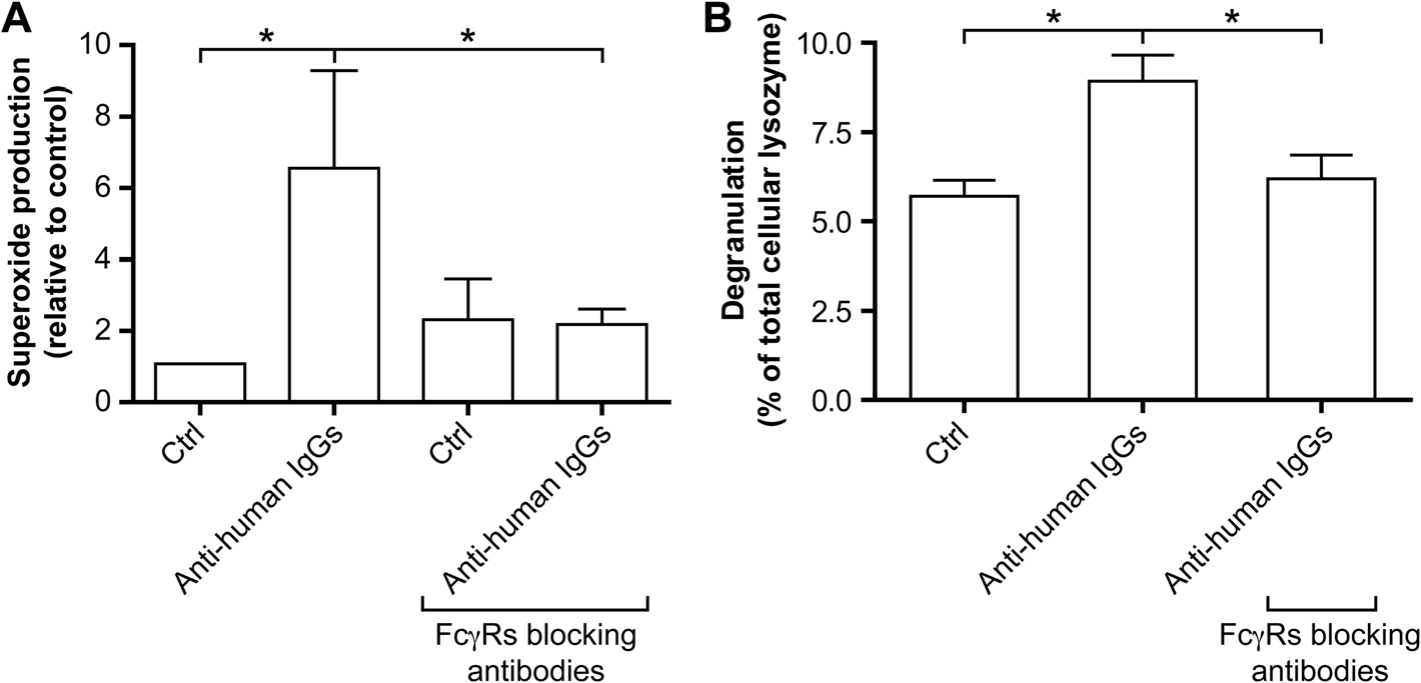
**Cross-linking of IgGs bound to human neutrophils leads to the secretion of superoxide anions and of lysozyme.** Isolated neutrophils were incubated with 10% autologous PPP in the presence or absence of FcγRs blocking antibodies. **A**: Neutrophils were resuspended at 5x10^6^ cells/ml in HBSS containing 125 μM of cytochrome *c* and anti-human IgGs antibodies for 10 minutes at 37°C. The reaction was stopped by transfer on ice and the cells were removed by centrifugation. Results are expressed as mean +/− SEM of three independent experiments. * = P < 0.05 (Wilcoxon signed rank test). **B**: Anti-human IgG antibodies were added for 30 minutes and the neutrophil supernatants were prepared as described in Material and Methods. Lysozyme release and superoxide production were quantified as described in Methods. Results are expressed as mean +/− SEM of three independent experiments. * = P < 0.05 (Mann–Whitney test).

The release of granule-contained proteolytic enzymes and the production of radical oxygen species are among the major means by which neutrophils destroy pathogens. However, these two functions will be deleterious for the cellular environment if misdirected (e.g., if these products are released into the extracellular milieu). As circulating neutrophils are continuously in contact with the vascular endothelium, we asked whether, in the context of RF-associated pathologies, cross-linking of surface-bound IgGs on neutrophils could trigger functions which impact on the integrity of the endothelium layer. It is relevant in this context to note that the extracellular release of lysozyme and superoxide anions has been described in response to the activation of neutrophils’ FcγRs [33-35]. We, therefore, examined these two functions in response to incubation with anti-human IgG antibodies. Cross-linking of surface-bound IgGs led to a 1.5 fold increase of the release of lysozyme and a 6 fold increase of the production of superoxide anions by neutrophils in suspension over that of unstimulated cells (Figure 3). Both functions were inhibited in the presence of anti-FcγRs antibodies. These results illustrate the capacity of circulating neutrophils to release toxic compounds in the extracellular milieu when present in conditions ressembling those present in RA.

**Figure 4:**
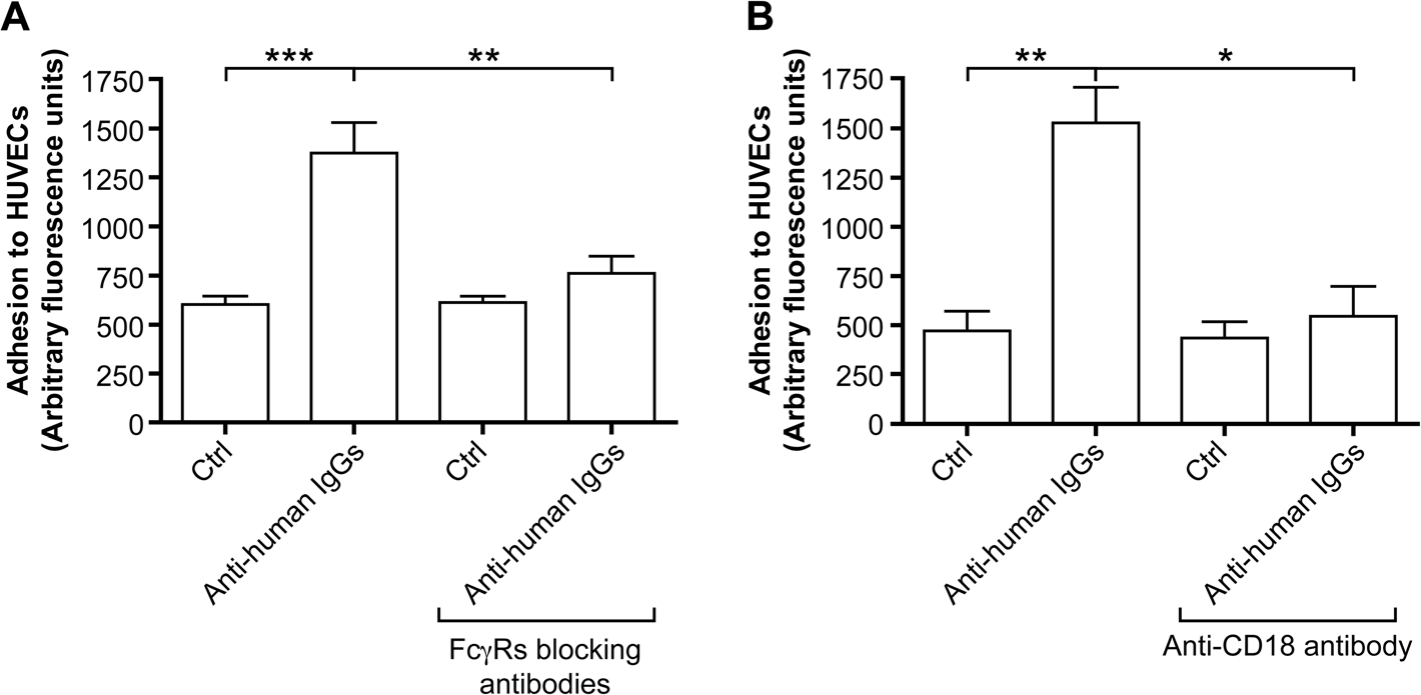
**Cross-linking of surface-bound IgGs leads to the adhesion of neutrophils to endothelial cells.** HUVECs were grown in 96 well plates until confluence. Neutrophils were incubated with calcein and with or without FcγRs blocking antibodies **(A)** or anti-CD18 antibody [37] (10 μg/ml) **(B)** before addition of PPP, as described in Methods. Neutrophils were then added to endothelial cells in the presence of anti-human IgG antibodies for 30 minutes. Non adherent neutrophils were discarded and the amount of adherent cells was quantified as the amount of fluorescence bound to endothelial cells. Results are expressed as mean +/− SEM of three independent experiments. * = P < 0.05, ** = P < 0.005, *** = P < 0.0005 (Mann–Whitney test).

Adhesion of neutrophils to endothelial cells is required in the context of innate immunity as neutrophils leave the vasculature to reach the inflammatory site. In the aseptic context of RA where blood-borne neutrophils encounter circulating ICs, we postulated that neutrophils will also be stimulated to adhere to the endothelium since activation of FcγRs leads to an increase of the expression and activation of adhesion molecules on neutrophils [36]. We tested this hypothesis by measuring the adherence of neutrophils to HUVECs. Calcein-labelled neutrophils were stimulated and incubated with endothelial cells for 30 minutes. We observed an increased capacity of stimulated neutrophils to adhere to endothelial cells (Figure 4). The adhesion is inhibited when neutrophils were pre-incubated with blocking anti-FcγR (Figure 4A) or anti-CD18 (Figure 4B) antibodies. Although the influence of blood flow is not considered in this static assay, this result indicates that cross-linking of IgGs bound on neutrophils leads to a β_2_-dependent adhesion of neutrophils to endothelial cells.

**Figure 5:**
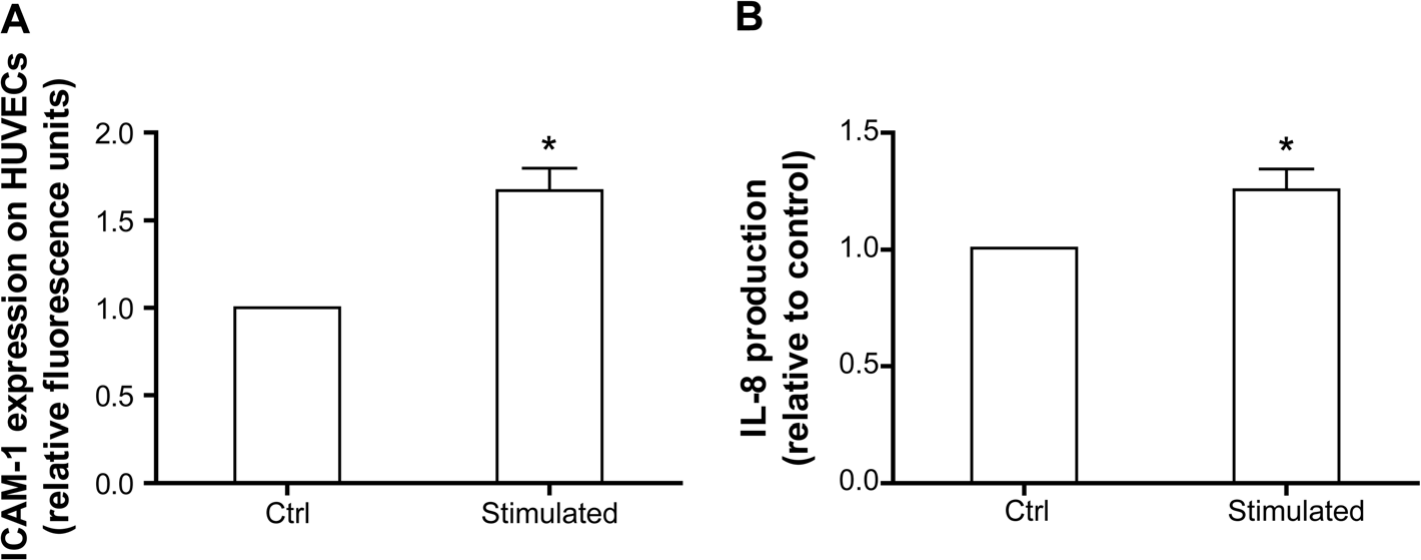
**ICAM-1 expression and IL-8 production in HUVECs.** HUVECs were incubated for 48 hrs with supernatants of control (Ctrl) or IgGs cross-linked human neutrophils (Stimulated) prepared as described in Material and Methods. **A**: The presence of ICAM-1 on HUVECs was measured by flow cytometry. HUVECs were washed with PBS and detached by 5 minutes incubation with accutase at 37°C. Cells were centrifuged, resuspended in HBSS/0.005% BSA and incubated with anti-ICAM-1 antibody (10 μg/ml) for 1 hour at 4°C before wash. They were then incubated with the secondary anti-mouse FITC-conjugated antibody (1/100) for 30 minutes at 4°C, washed and fluorescence was analysed. **B**: IL-8 release by HUVECs was measured by ELISA. The supernatants of HUVECs were collected and the remaining cells were discarded by centrifugation. The amount of IL-8 in the supernatants was measured in duplicates using the human IL-8 CytoSet from Invitrogen (catalog no.CHC1303) following the manufacturer’s instructions. IL-8 contained in neutrophils supernatants was substracted. Results are expressed as mean +/− SEM of three independent experiments. * = P < 0.05 (one-sample t test).

The results presented in Figure 3 show that neutrophils release superoxide anions and lysozyme into the extracellular medium subsequently to the cross-linking of surface-bound IgGs. Because of the capacity of stimulated neutrophils to adhere to endothelium (Figure 4), these mediators will be concentrated in the endothelial microenvironment. Accordingly, we next decided to examine the capacity of FcγRs-activated neutrophils to modulate the phenotype of endothelial cells. As we observed a β_2_ integrin-dependent increase in the adhesion of neutrophils subsequently to FcγR cross-linking, we examined the expression on the endothelial cells of the adhesion molecule ICAM-1, the counterpart of β_2_–integrins whose expression on endothelial cells is increased in response to inflammatory mediators [38]. We observed that ICAM-1 expression is increased when HUVECs were incubated for 48 hours with the supernatants of stimulated but not of control neutrophils (Figure 5A). We also measured the production of IL-8 by HUVECs as this chemokine plays a major role in neutrophil adhesion and migration. HUVECs were incubated with neutrophil supernatants prepared as described above and IL-8 was measured by ELISA. We observed a slight but significant increase of soluble IL-8 production by HUVECs exposed to supernatants of stimulated neutrophils (Figure 5B). The amounts of IL-8 measured are likely to represent an underestimate of the actual amounts produced as secreted IL-8 by HUVECs will bind to extracellular aminoglycans residues of these cells and therefore will be undetectable by ELISA [38,39]. The release of IL-8 by stimulated and unstimulated neutrophils was also measured and the values were substracted from the amounts measured in the supernatants of HUVECs.

**Figure 6:**
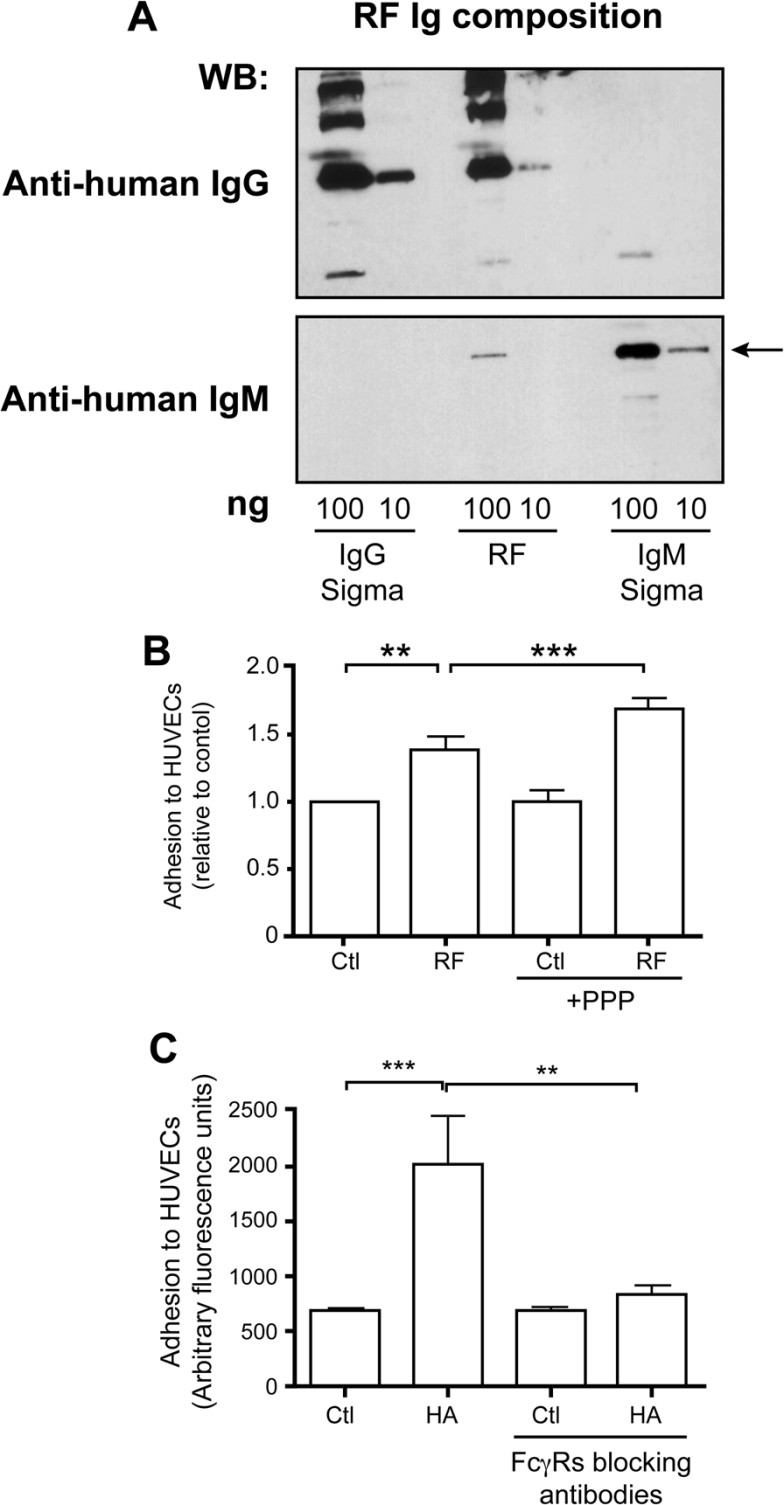
**Rheumatoid factors induce FcγRs-dependent adhesion of neutrophils to HUVECs. A**: An aliquot of the preparation of rheumatoid factors (100 ng and 10 ng) was analyzed by SDS-PAGE followed by immunoblotting with horseradish peroxidase-labelled anti-human IgG or anti-human IgM antibodies. The same amounts of commercial human IgG and IgM were analyzed on the same gel. The IgM band was used for quantification (arrow). **B**: Neutrophils were incubated with calcein and with or without PPP, as described in Methods. Neutrophils were then plated on a confluent monolayer of endothelial cells and RFs (500 μg/ml) were added for 2 hours. Non-adherent neutrophils were discarded and the amount of adherent cells was quantified as the amount of fluorescence bound to endothelial cells. Results are expressed as mean +/− SEM of three independent experiments. ** = P < 0.005, *** = P < 0.0005 (one-sample t test). **C**: Heat aggregated IgGs (HA-IgGs) were prepared as previously described [41]. Neutrophils were incubated with calcein, plated on endothelial cells and 100 μg/ml HA-IgG (HA) were added for 2 hours. Results are expressed as mean +/− SEM of three independent experiments, each carried out in triplicate. ** = P < 0.005, *** = P < 0.0005 (Mann–Whitney test).

Although the presence of RFs is associated with the severity of RA symptoms, very few studies have defined a biologic or pathologic function of these antibodies [40]. The results presented above suggest that RFs either as ligands of FcγRs or as antibodies recognizing membrane-bound IgGs might activate neutrophils. To verify this hypothesis, we decided to test the capacity of human RFs to induce neutrophil adhesion to HUVECs as this experiment is performed in 96-well plates and consequently requires low amounts of RF. We first characterized the immunoglobulin composition of the RF preparation. An aliquot was submitted to SDS-PAGE followed by immunoblotting with anti-human IgG or anti-human IgM. We observed that this RF preparation is an IgM/IgG complex containing 5% IgM and 95% IgG, as estimated by densitometry (Figure 6A). Thus, the complexes collected using 5% PEG are the IgM-RFs bound to their cognate IgG antigen. Being a pentamer, IgM-RFs preferentially form immune complexes in the presence of IgGs. As the methods used to dissociate IgMs from IgGs (acid, high salt) can cause denaturation of the RF with resulting loss of binding activity, we used in the subsequent experiments the native RF preparation as so. We incubated neutrophils with the RF complexes and monitored their adhesion to HUVECs as in Figure 4. To assess that RF can bind membrane-bound IgGs, we performed these experiments without or with a pre-incubation of neutrophils with PPP on the adhesion capacity (see Figure 1). We observed an increase of adhesion to HUVECs when neutrophils were incubated with 500 μg/ml of purified RF and this adhesion was enhanced when neutrophils were pre-incubated with PPP (Figure 6B). This observation confirms the hypothesis that RFs can bind IgGs on neutrophils leading to cell activation. Adhesion was not modified in the presence of commercial non-specific IgM alone (data not shown). The relatively high concentration of RFs required for observing a functional effect when compared with the commercial anti-human cross-linking antibodies (20 μg/ml) is explained by the low concentration of IgM-RFs present in the preparation. Furthermore, as the affinity of most IgM-RFs for IgG is relatively low (of the order of 10^5^ to 10^7^) there is a constant on/off binding of the IgG-RF to IgGs or FcγR-bound IgGs. As we showed that the RF preparation was an IgG-containing complex, we tested the capacity of IgG complexes to enhance adhesion. Incubation of neutrophils with heat-agregated IgGs (HA-IgGs, 100 μg/ml) enhances their adhesion to HUVECs in an FcγRs-dependent manner (Figure 6C). Taken together, these results indicate that RFs from RA patients can activate neutrophils by recognizing either IgGs or FcγRs on neutrophils leading to an increased adhesion to endothelium.

## Discussion

The mechanisms neutrophils use to kill microorganisms have the potential to alter or injure normal tissues [27]. Neutrophils’ Fcγ receptors illustrate this paradox. The best described role for neutrophils’ FcγRs is the optimization of the phagocytosis of IgG-opsonized pathogens [42-46]. However, these receptors are also involved in a variety of IgG-dependent processes including clearance of the naturally occurring immune complexes and the pathogenesis of auto-immune disorders such as RA [13]. Although RFs are present in the serum of the majority of RA patients and are a predictor of disease severity, their potential role in FcγRs activation and in RA pathogenesis is poorly characterized [47,48]. Yet, as IgG-IgM complexes (see Figure 6), RFs complexes represent potential ligands for FcγRs or for IgGs bound to FcγRs.

We observed that incubation of neutrophils with a preparation of RFs enhanced their ability to adhere to HUVECs. To our knowledge, this result represents the first observation of a biological effect of human RFs which could participate in the RA-associated vascular inflammation. In addition, we showed that activation of FcγRs of neutrophils with anti-human IgG antibodies which mimick the presence of RFs leads within 30 minutes to an extracellular release of lysosyme and superoxide anions concomitant with an enhancement of the adhesion of neutrophils to endothelial cells. These events may end up damaging the endothelium, a possibility supported by our observation that supernatants of neutrophils in which FcγRs have been cross-linked altered the phenotype of endothelial cells in an inflammatory direction (increased expression of ICAM-1 and production of IL-8). As IL-8 is a potent chemotactic mediator for neutrophils, the combined increase of IL-8 and ICAM-1 will lead to a positive activation loop of recruitment and adhesion of neutrophils with the potential to induce local vascular injury.

Few studies have characterized blood neutrophils of RA patients in spite of the demonstrated presence of circulating IC. Chronic peripheral activation of neutrophils in polyarticular juvenile RA has been reported and data suggest that blood neutrophils from RA patients have impaired FcγR-dependent bactericidal functions (superoxide production) [49,50]. On the other hand, generation of mice with neutrophil-selective expression of FcγRIIa and FcγRIIIb indicate that, in a model of soluble immune complexes deposited in the vasculature, FcγRIIIb is responsible for slowing down neutrophils thereby facilitating adhesion to endothelial cells [13,26]. Immobilized neutrophils may then activate FcγR-dependent cytotoxic functions and promote local endothelium damage. In innate immunity, by contrast, the vascular wall is rarely in contact with the toxic products of neutrophils as neutrophils leave the circulation by transendothelial migration. They arrive at the inflammation site where they release radical oxygen species and granular contents, most often in phagosome, thus preventing major damage to the cellular environment.

In humans, a similar mechanism of activation of FcγRs on circulating neutrophils has previously been described in anti-neutrophil cytoplasm autoantibody (ANCA)-associated diseases. These antibodies activate circulating neutrophils leading to vascular inflammation, a process in which FcγRs are directly involved. ANCAs are directed against enzymes stored in neutrophil granules but which have translocated to the plasma membrane in response to a minor inflammatory stimulus. The F(ab’)_2_ portion of ANCAs engages the antigen and the Fc portion engages FcγRs. This FcγR-mediated interaction is required for neutrophil activation [51,52]. This mode of stimulation of neutrophils by ANCAs closely mimicks the mode of stimulation of RF proposed in the present study and argues for a role of RFs and RF-containing IC in RA-associated vascular inflammation. ANCAs-associated diseases, such as Wegener granulomatosis, clearly indicate that activation of circulated neutrophils can lead to vascular inflammation.

We showed that the circulating neutrophils possess membrane-bound IgGs which may be recognized by RFs leading to their activation. The mechanism of action of RF may require both the direct recognition of RF complexes by free FcγRs, such as HA-IgG and the binding of membrane-bound IgGs by RFs as pre-incubation of neutrophils with PPP enhances the RF-dependent activation of neutrophils.

Several data suggest that the affinity of FcγRIIa may be modulated by a priming signal such as fMLP, TNF or a mechanism including granular proteases [53,54]. This is of particular relevance in the inflammatory context of arthritis where elevated concentrations of various cytokines and mediators are present and may activate circulating neutrophils. A cytokine-dependent increase of FcγRs affinity would influence the amount of membrane-bound IgGs and consequently the RF-dependent activation of neutrophils of RA patients. Of direct relevance to the present study, the efficiency of anti-TNF therapy is related to a loss of function of FcγRIIa [21]. This effect may be mediated by the ability of TNF to modulate the functional responsiveness of FcγRIIa [55] and may, at least in part, explain the positive effects of anti-TNF therapy on endothelial dysfunction as previously proposed [56].

In the present study, we used a RF preparation purified from the sera of RA patients. This RF containing preparation is polyclonal, i.e., that it contained different populations of RFs (IgM and possibly IgG) with different specificities and affinities. The affinity of RFs for IgGs combined with the modulation of FcγR affinity for IgG by RA-associated cytokines will impact on the FcγR-dependent activation of neutrophils and consequently on the inflammation of the endothelium observed in RA patients. Further experiments using neutrophils from RA patients untreated or treated with anti-TNF therapy should help to understand not only the role of RA-associated cytokines on FcγR activity, but also the role of RF in the pathogenesis of RA.

In RA patients, high concentrations of RFs and the presence of extra-articular manifestations such as vasculitis are predictors of increased mortality, primarily from cardiovascular diseases [57]. Patients with ANCA-associated diseases (Wegener granulomatosis) also present higher risks to develop cardiovascular diseases [58]. On the other hand, FcγRs have been proposed to play a critical role in atherogenesis. Indeed, in apoE−/−mice (a model of human atherosclerosis), FcγRs appear to be important for the clearance of LDL-containing IC and apoE−/− mice deficient for the activating γ chain of FcγRs show less development and progression of atherosclerosis than apoE^−/−^ mice which possess the γ chain of FcγRs [59]. These data support a role for leukocytes’ FcγRs in cardiovascular diseases associated with auto-immune diseases.

Systemic lupus erythematosus (SLE) is a chronic inflammatory disease also characterized by systemic inflammation and the presence of auto-antibodies and RF. Patients with SLE have an increased mean intima media thickness and SLE represents a risk factor for cardiovascular disease [60,61]. This indicates that the engagement of FcγRs of neutrophils and other immune cells may be involved in vascular dysfunction associated with various auto-immune inflammatory diseases.

Further examinations of neutrophil responses to RFs isolated from different patients are warranted. Indeed, the Ig composition, the affinity and the size of the RF complexes could influence neutrophil activation. Nevertheless, our results present a first report underlining the role of RFs in FcγR-dependent activation of circulating neutrophils and the potential consequences on systemic vascular inflammation. We previously characterized several of the pathways downstream of neutrophil’s FcγRs and we showed that both receptors cooperate for optimal FcγR-dependent responses [62,63]. In particular, FcγRIIIb activation triggers a specific calcium signal [41]. As FcγRIIIb is nearly exclusively expressed on human neutrophils, targeting this receptor or the FcγRIIIb-dependent calcium signal, may represent an interesting clinical avenue which could help control RF-associated systemic inflammation in RA patients.

## Conclusions

We show here that rheumatoid factors either by recognizing membrane-bound IgGs or by direct binding to FcγRs can activate circulating neutrophils. As this activation leads to adhesion to endothelial cells and may modify the phenotype of endothelial cells, we propose that it participates in the rheumatoid arthritis-associated vascular damages.

## Abbreviations

ANCA: Anti-neutrophil cytoplasm autoantibody; FcγR: Fcgamma receptor; fMLP: *N*-formyl-methionyl-leucyl-phenylalanine; HUVECs: Human umbilical vein endothelial cells; IC: Immune complex; ICAM-1: Intercellular adhesion molecule-1; PPP: Platelet-poor plasma; RA: Rheumatoid arthritis; RF: Rheumatoid factors.

## Competing interests

The authors declare that they have no competing interests.

## Authors’ contributions

ERL designed the study, performed the experiments, analysed the data and wrote the manuscript. MV performed several experiments and analysed data and statistics. LM participated in the design of the study and in the analysis of the data. MMN participated in the design of the study and provided material and expertise with RF. PEP revised the manuscript and provided clinical expertise and helpful discussions. PHN designed the study, critically analysed the data and supervised the preparation of the manuscript. All the experiments were performed in PHN’s laboratory. All authors read and approved the final manuscript.
